# Characterization of STAT3 expression, signaling and inhibition in feline oral squamous cell carcinoma

**DOI:** 10.1186/s12917-015-0505-7

**Published:** 2015-08-14

**Authors:** Megan E. Brown, Misty D. Bear, Thomas J. Rosol, Chris Premanandan, William C. Kisseberth, Cheryl A. London

**Affiliations:** Department of Veterinary Clinical Sciences, College of Veterinary Medicine, The Ohio State University, Columbus, OH USA; Department of Veterinary Biosciences, College of Veterinary Medicine, The Ohio State University, Columbus, OH USA

**Keywords:** STAT3, Oral squamous cell carcinoma, Feline

## Abstract

**Background:**

Signal transducer and activator of transcription 3 (STAT3) plays a critical role in tumor development by regulating signaling pathways involved in cell proliferation, survival, metastasis and angiogenesis. STAT3 is activated in many cancers, including head and neck squamous cell carcinoma (HNSCC) in people. Feline oral squamous cell carcinoma (OSCC) is similar to advanced or recurrent HNSCC as it is poorly responsive to traditional therapies and carries a poor long-term prognosis. The purpose of this study was to characterize expression and activation of STAT3 in feline OSCC cell lines and tumor samples and to investigate the biologic activity of a novel, allosteric STAT3 inhibitor, LLL12, in feline OSCC cell lines.

**Results:**

We evaluated 3 feline OSCC cell lines and one of these (SCCF2) exhibited high levels of constitutive STAT3 phosphorylation and high sensitivity to LLL12 treatment. Exposure of SCCF2 cells to LLL12 resulted in decreased expression of pSTAT3 and total STAT3, apoptosis as assessed by caspase 3/7 activation, inhibition of colony formation and reduced expression of the STAT3 transcriptional target survivin. In contrast, the STAT3 transcriptional targets VEGF and MCL-1 increased after LLL12 treatment. This was, in part, likely due to LLL12 mediated upregulation of HIF-1α, which is known to drive VEGF and MCL-1 expression. The OSCC cell lines with low basal STAT3 phosphorylation did not exhibit these effects, suggesting that STAT3 inhibition was responsible for the observed results. Lastly, immunohistochemistry for pSTAT3 was performed using a feline OSCC tissue microarray, demonstrating expression in 48 % of samples tested.

**Conclusions:**

These data demonstrate that LLL12 has biologic activity against a feline OSCC cell line expressing pSTAT3 and that STAT3 represents a target for therapeutic intervention in this disease. However, given the up-regulation of several STAT3 transcriptional targets following treatment, further investigation of STAT3 and its related signaling pathways in OSCC is warranted.

**Electronic supplementary material:**

The online version of this article (doi:10.1186/s12917-015-0505-7) contains supplementary material, which is available to authorized users.

## Background

The signal transducer and activator of transcription (STAT) family of proteins consists of seven isoforms that are transcription factors responsible for relaying signals from extracellular growth factors and cytokines, thereby influencing normal cellular physiology and development. Due to their role in cell survival, proliferation and angiogenesis, STAT proteins, in particular STAT3 and STAT5, also have been implicated in cancer initiation and progression [1]. Initial evidence of the role of STAT proteins in tumor biology came from the discovery that activated STAT3 was required for cellular transformation by the viral oncogene, v-src [1, 2]. There is now substantial data supporting the role of STAT proteins in cancer biology, including the observation that constitutive activation of STAT3 is present in a variety of human malignancies [1, 3].

While phosphorylation of STAT3 was initially believed to be primarily driven by the Janus kinase (JAK) family members following cytokine receptor signaling, it is now evident that substantial cross-talk exists between receptor and non-receptor kinases (e.g. EGFR, Src), and this also contributes to the activation of STAT3 [4, 5]. After phosphorylation of the critical tyrosine residue (Tyr705), STAT3 homodimerization occurs followed by nuclear translocation and alteration of gene transcription. In normal cells, STAT3 activation is transient due to endogenous regulating proteins including SH2-containing phosphatases (SHPs), protein inhibitors of activated STAT3 (PIAS3) and suppressors of cytokine signaling (SOCS) [6]. Since activating mutations of STAT3 are rare [7], constitutive phosphorylation of STAT3 is generally the result of aberrations in upstream signaling proteins or physiologic regulators [5, 8].

Importantly, constitutive STAT3 activation contributes to metastasis and chemotherapy resistance through its effects on several transcriptional targets, including VEGF, survivin and Bcl2 family members. As such, it represents a potentially relevant target for therapeutic intervention in many cancers and several inhibitors of STAT3 are currently under investigation [9, 10]. Recently, the role of STAT3 in human head and neck squamous cell carcinoma (HNSCC) has received significant attention as constitutive activation has been demonstrated in several HNSCC cell lines [11, 12]. Inhibition of STAT3 with dominant negative constructs or antisense oligonucleotides promoted downregulation of expression of STAT3 transcriptional targets, growth inhibition and induction of apoptosis in HNSCC cell lines, suggesting that STAT3 is necessary for growth and survival of HNSCC cells [11]. Similar results were generated in HNSCC murine xenograft models treated with STAT3 decoys or siRNA [13]. Lastly, STAT3 phosphorylation was found in HNSCC tumor samples and has been associated with a poor prognosis [14].

Similar to people, squamous cell carcinoma (SCC) is the most common oral malignancy in cats, representing 61 % of oral tumors [15]. Feline oral SCC (OSCC) is locally invasive, rapidly progressive and poorly responsive to traditional therapies such as radiation therapy and chemotherapy. In most cases, OSCC arising from the gingiva exhibits bone invasive properties early in the course of disease, causing substantial morbidity in the form of anorexia and pain. Furthermore, as OSCC primarily occurs in older cats, it is often mistaken for dental disease resulting in advanced tumor stage at the time of diagnosis. The overall prognosis for cats diagnosed with OSCC is poor, with a one year survival rate of less than 10 % [16]. In rare cases, surgery followed by radiation therapy may provide adequate tumor control in patients with small tumors or tumors located in the rostral mandible [17]. However, recurrence rates following surgery are generally considered unacceptably high and acute and long-term post-surgical morbidities are common [17, 18]. Radiation therapy is also considered ineffective in management of OSCC as both definitive and hypofractionated protocols fail to extend survival beyond 2–3 months in most instances [19, 20]. Finally, chemotherapy alone has minimal benefit in treatment of OSCC [21]; however, the combination of radiation therapy and chemotherapy or radiosensitizers results in modest improvements in response rates and outcomes [22] but use is limited by increased local and systemic toxicity [23].

Given the similarities between feline and human SCC and the paucity of effective treatments in cats with this disease, we sought to determine whether STAT3 plays a role in feline OSCC. In previous studies we have shown that the allosteric, small molecule inhibitor, LLL12, is a potent and selective inhibitor of STAT3 phosphorylation [24]. Specifically, LLL12 reduces STAT3 phosphorylation, inhibits proliferation, and induces apoptosis in a variety of canine and human cell lines (breast, pancreatic, glioblastoma, osteosarcoma) and inhibits tumor growth and angiogenesis in several murine tumor xenograft models [24–26]. As such, the purpose of this study was to characterize activation and expression of phosphorylated STAT3 (pSTAT3) in feline OSCC cell lines and tumor samples and to investigate the biologic activity of LLL12 in feline OSCC cell lines.

## Methods

### Cell lines and reagents

Feline OSCC lines SCCF1, SCCF2 and SCCF3 were previously established and characterized by one of the investigators (TJR) [27, 28]. The SCCF1, SCCF2 and SCCF3 cell lines were derived from a laryngeal, bone-invasive gingival and lingual SCC, respectively. Cell lines were maintained in DMEM supplemented with 10 % FBS, non-essential amino acids, sodium pyruvate, penicillin, streptomycin, L-glutamine, and HEPES (4-(2-hydroxythyl)-1-piperazineethanesolfonic acid) at 35 °C, supplemented with 5 % CO_2_. LLL12 was synthesized and purified as previously described and provided by Dr. Chenglong Li (College of Pharmacy, The Ohio State University) [24]. Aliquots of the stock solution were stored at -20 °C until use. The proteasome inhibitor MG132 was purchased from Calbiochem (Billerica, MA). The following antibodies were used for Western blotting and/or immunohistochemistry experiments: pSTAT3 (Y705, Cell Signaling Technology, Danvers, MA), STAT3 (Cell Signaling Technology), survivin (Novus Biologicals, Littleton, CO), pAKT (Ser473, Cell Signaling Technology), AKT (BD Biosciences, San Jose, CA) and β-actin (Santa Cruz Biotechnology, Santa Cruz, CA).

### Cell proliferation

Optimal 96-well plating densities were determined for each cell line. This was defined as the concentration of cells at which proliferation occurred but cell confluence and/or medium color change did not occur in 48 h. Feline OSCC cells (SCCF1: 1 × 10^4^; SCCF2: 1 × 10^4^; SCCF3: 5 × 10^3^) were seeded in triplicate in 96-well plates overnight in 10 % FBS supplemented medium and incubated with DMSO or increasing concentrations of LLL12. After 24 h, the medium was removed and the plates were frozen at -80 °C overnight before processing using the CyQUANT® Cell Proliferation Assay Kit (Molecular Probes, Eugene, OR) according to the manufacturer’s instructions. Cell proliferation was calculated as a percentage of the DMSO-treated control wells and IC_50_ values were derived after plotting proliferation values on a logarithmic curve.

### Detection of apoptosis

Feline OSCC cells (SCCF2: 1 × 10^4^; SCCF3: 5 × 10^3^) were seeded in triplicate in 96-well plates overnight in 10 % FBS supplemented medium and incubated with DMSO or increasing concentrations of LLL12 for 12 or 24 h. Caspase 3/7 activity was determined using the SensoLyte Homogenous AMC Caspase 3/7 Assay (Anaspec Inc, San Jose, CA) according to the manufacturer’s instructions.

### Clonogenic assay

SCCF2 and SCCF3 cells were grown in flasks until approximately 80 % confluent, then collected, washed and plated at 1,000 cells per well in six-well plates in 10 % FBS supplemented medium. After 24 h, the cells were treated with DMSO, 0.02, 0.2 or 2 μM LLL12 and incubated at 35 °C, supplemented with 5 % CO_2_ until colony formation was observed in DMSO treated control wells. Cells were then fixed with methanol/acetic acid (3:1), washed with PBS, stained with crystal violet (0.2 g/L) and colonies consisting of greater than 50 cells were counted. After counting colonies, plating efficiency and survival fraction were calculated. Plating efficiency was defined as the number of colonies formed divided by the number of cells seeded in DMSO treated groups. Survival fraction was defined as the number of colonies formed divided by the number of cells seeded in LLL12 treated groups, normalized to the plating efficiency [29].

### Western blotting

Cell lines were cultured in 10 % FBS supplemented medium until 80 % confluent. Protein lysates were prepared and quantified, 100 μg of protein was separated by SDS-PAGE and Western blotting for pSTAT3 and STAT3 was performed as previously described [30]. To evaluate effects of LLL12 treatment, SCCF2 and SCCF3 cells (1 × 10^7^) were cultured in 1 % FBS supplemented medium and treated with DMSO, 0.2 μM LLL12 alone, 10 μM MG132 alone or MG132 in combination with LLL12 for 12 h. Protein lysate preparation, quantification and Western blotting were repeated as described above. The membranes were incubated overnight with anti-pSTAT3, anti-survivin or anti-pAKT antibodies incubated with the appropriate horseradish peroxidase-linked secondary antibodies, washed, and exposed to substrate (SuperSignal West Dura Extended Duration Substrate, Pierce, Rockford, IL). Blots were stripped, washed, and re-probed for total STAT3, AKT or β-actin.

### RT-PCR and qRT-PCR

Total RNA was extracted from SCCF2 and SCCF3 cells cultured in 1 % FBS supplemented medium following 12 h of treatment with DMSO or 0.2 μM LLL12, using TRIzol reagent (Invitrogen, Carlsbad, CA). cDNA was made from 2 μg of total RNA using Superscript III (Invitrogen) according to the manufacturer’s instructions. For each PCR reaction, 1/20th of the resultant cDNA was used in a total volume of 25 μL. Primers designed and utilized for feline survivin, VEGF, STAT3, HIF-1α, cyclin D1, MCL-1 and GAPDH are listed in Additional file 1: Table S1. An annealing temperature of 60 °C was used for all reactions. Standard PCR was performed with all primer sets and amplicon length was verified through agarose gel electrophoresis and visualization of PCR products using the Alpha Imager system (Alpha Innotech Corp, San Leandro, CA).

To quantitatively measure the effect of LLL12 treatment on STAT3 gene expression as well as downstream targets, total RNA was collected using the methods described above. Real-time quantitative PCR was performed using Applied Biosystem’s StepOne Plus Real-Time PCR system (Applied Biosystems, Foster City, CA). Feline survivin, VEGF, STAT3, HIF-1α, cyclin D1, MCL-1 and GAPDH were detected using Fast SYBR Green PCR Master Mix (Applied Biosystems) according to the manufacturer’s instructions. All reactions were performed in triplicate and included non-template controls for each gene. Relative expression was calculated using the comparative threshold cycle method. With this method, the cycle threshold (*C*_T_) values are compared between the sample of interest and a non-treated sample. The *C*_T_ values of both samples are normalized to an endogenous housekeeping gene, GAPDH [31].

### Tissue microarray construction and immunohistochemistry

To assess the prevalence of STAT3 activation in primary feline OSCC samples, a tissue microarray (TMA) was made from archived feline OSCC surgical biopsies and immunohistochemistry for pSTAT3 was performed. Surgical biopsies were obtained and evaluated microscopically to confirm the presence of OSCC as previously described [32]. Representative areas of tumor tissue were identified on hematoxylin and eosin (HE) stained sections by a single pathologist (CP) for 1.5 mm core sampling. Cores were extracted from the corresponding areas on thirty-seven paraffin embedded tissue blocks and inserted into predetermined sites on the TMA recipient block. Immunohistochemical staining was performed for pSTAT3 (Cell Signaling Technology). Briefly, slides containing tissue sections were deparaffinized in xylene and rehydrated in distilled water. Slides were then placed in plastic coplin jars containing Target Retrieval Solution (Target Retrieval Solution, pH 6.0, Dako, Glostrup, Denmark) and then enclosed in a decloaking chamber containing distilled water. Antigen retrieval was performed by heating the slides to 125 °C for 15 min; slides were allowed to cool to 90 °C for 10 s followed by cooling at room temperature for 10 min. Endogenous peroxidase activity was quenched by immersion in 3 % hydrogen peroxide for 10 min. Non-specific binding was blocked by incubation with serum-free protein block (Dako) for 10 min. Slides were incubated for 30 min at room temperature with anti-pSTAT3 antibody at a dilution of 1:100 in antibody diluent (Dako). After rinsing in wash buffer, biotinylated goat-anti-rabbit secondary antibody was applied at a dilution of 1:200 for 30 min. All slides were rinsed and then incubated with Vector RTU ABC Elite complex (Vector Laboratories, Burlingame, CA) for 30 min followed by incubation with DAB chromagen for 5 min. Finally, slides were counterstained with hematoxylin, rinsed, dehydrated with ethanol and fitted with coverslips. For negative controls, staining procedures were carried out as above but without the addition of a primary antibody. Mouse lymph node served as a positive control. The construction of the tissue microarray and immunohistochemistry were performed by the OSU-CVM Veterinary Biosciences Comparative Pathology and Mouse Phenotyping Shared Resource Histology/IHC Core Lab.

Slides were evaluated by light microscopy to assess pSTA3 immunoreactivity. Overall pSTAT3 signal intensity of OSCC cells was subjectively scored from 0 to 3 (0 = none to weak, 1 = mild, 2 = moderate, 3 = strong), with strong reactivity defined as staining intensity of the positive control. The percentage of positive cells was also estimated and scored as follows: <5 % = 0, 5–20 % = 1, 20–50 % = 2, 50–100 % = 3. A total score was obtained by adding the score of signal intensity and percentage positivity. Total scores greater than or equal to 2 were considered positive. The distribution of pSTAT3 immunoreactivity was noted as nuclear in both positive control and OSCC samples.

### Statistical analysis

All experiments were performed in triplicate and/or repeated three times. All values reported are mean ± standard deviation. A one-way ANOVA comparison (parametric data) or Kruskal-Wallis test (non-parametric data) was performed to compare multiple treatment groups in the cell proliferation, caspase and colony formation assays. An independent samples t-test (parametric data) or Mann–Whitney U test (non-parametric data) was performed to evaluate differences in gene expression between LLL12 treated groups and DMSO treated controls for real-time quantitative PCR assays. Values of p < 0.05 were considered statistically significant.

## Results

### Constitutive phosphorylation of STAT3 is variable in feline OSCC cell lines and correlates with response to LLL12

**Fig. 1:**
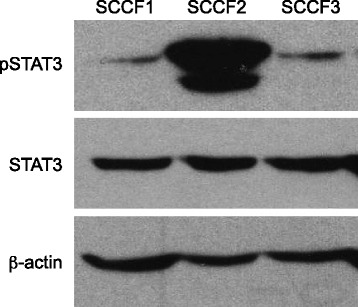
Activation of STAT3 in feline OSCC cell lines. Protein lysates were generated from the three untreated cell lines, separated by SDS-PAGE and Western blotting for pSTAT3 (Y705), STAT3 and β-actin was performed

**Fig. 2:**
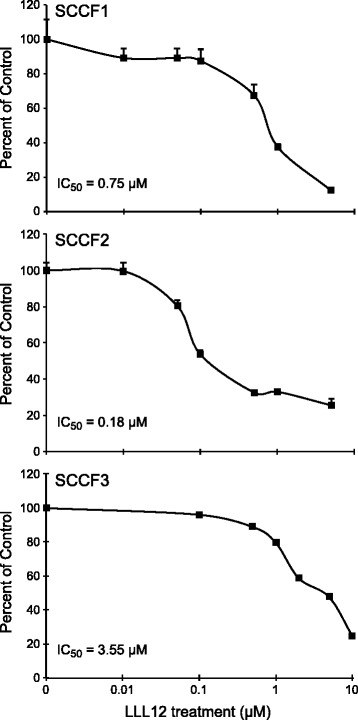
Effects of LLL12 on proliferation in feline OSCC cell lines. Feline OSCC cell lines were treated with DMSO or LLL12 at increasing concentrations (0.01-10 μM) for 24 h and proliferation was assessed using CyQUANT® Cell Proliferation Assay Kit. Experiments were performed in triplicate and IC_50_ values were calculated

To assess whether STAT3 is constitutively activated in feline OSCC cell lines, Western blotting was performed to evaluate for the presence of pSTAT3. As shown in Fig. 1, only one of the three cell lines, SCCF2, demonstrated high levels of pSTAT3 when compared to the others. All three OSCC cell lines were then incubated with increasing concentrations of LLL12 (0.01 μM – 10 μM) for 24 h and cell proliferation was assessed. While LLL12 significantly reduced cell proliferation in all cell lines (p < 0.005), SCCF2 was most sensitive to LLL12, with a calculated IC_50_ concentration in the nanomolar range (180 nM), correlating with the highest level of basal STAT3 phosphorylation (Fig. 2).

### Treatment with LLL12 inhibits pSTAT3 and STAT3 expression in feline OSCC cells that express elevated levels of pSTAT3

**Fig. 3:**
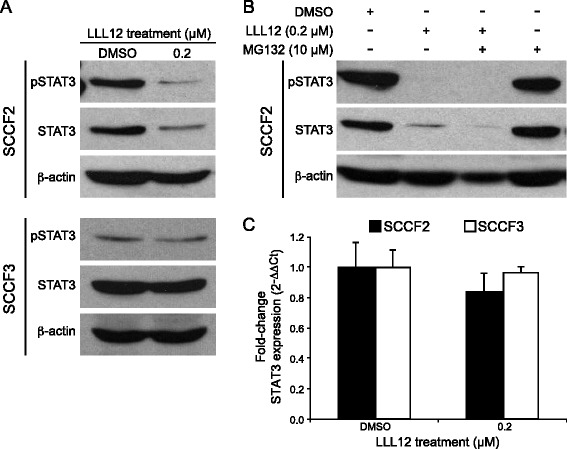
Effects of LLL12 treatment on pSTAT3 and STAT3 expression in feline OSCC cell lines. **a** Feline OSCC cell lines were treated with DMSO or 0.2 μM LLL12 for 12 h prior to collection. Protein lysates were generated, separated by SDS-PAGE and Western blotting for pSTAT3, STAT3 and β-actin was performed. **b** SCCF2 cells were treatment with 0.2 μM LLL12 alone, 10 μM MG132 alone or MG132 in combination with LLL12 for 12 h. **c** Feline OSCC cell lines were treated with DMSO or 0.2 μM LLL12 for 12 h. RNA was collected and quantitative RT-PCR for STAT3 was performed

To assess the direct effects of LLL12 on pSTAT3, two OSCC cell lines with high (SCCF2) and low (SCCF3) basal STAT3 activation were chosen for further analysis. Cells were treated with DMSO or 0.2 μM LLL12 for 12 h and Western blotting for pSTAT3 and STAT3 was performed. Western blot analyses revealed that pSTAT3 and, to a lesser degree, total STAT3, were both downregulated in SCCF2 following treatment with LLL12 (Fig. 3a). In contrast, no change in pSTAT3 or STAT3 expression was observed in the SCCF3 cell line.

As ubiquitin-mediated proteasomal degradation is a known regulatory mechanism for STAT family protein turnover [33, 34], SCCF2 cells were treated with the proteasome inhibitor, MG132, to determine if this mechanism was responsible for the loss of total STAT3. Cells were treated with LLL12 alone, MG132 alone or MG132 in combination with LLL12 for 12 h. Western blot analysis demonstrated that MG132 did not block LLL12 mediated downregulation of total STAT3, suggesting that ubiquitin-mediated proteosomal degradation is not the mechanism responsible for loss of total STAT3 following LLL12 treatment in feline OSCC cells (Fig. 3b). Lastly, because STAT3 mRNA expression levels did not decrease following LLL12 treatment, loss of total STAT3 was likely not due to autoregulation whereby pSTAT3 can enhance STAT3 gene transcription [35]. Although there was slight decrease in STAT3 mRNA following LLL12 treatment in the SCCF2 cell line, this difference was not statistically significant (Fig. 3c).

### Treatment with LLL12 promotes apoptosis of feline OSCC cells that express elevated levels of pSTAT3

**Fig. 4:**
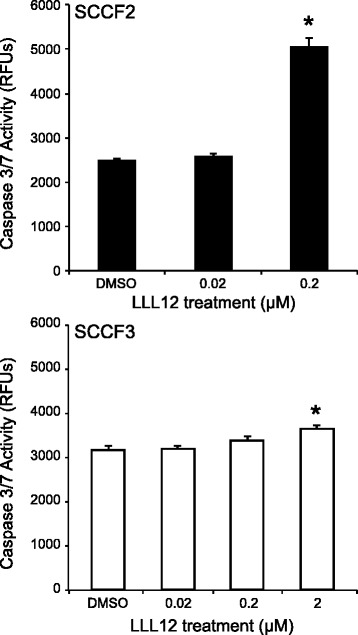
Evaluation of feline OSCC cell lines for apoptosis following LLL12 treatment. Feline OSCC cell lines were treated with DMSO or increasing concentrations of LLL12 (0.2 – 2 μM) for 24 h. Caspase activity was assessed using SensoLyte Homogenous AMC Caspase 3/7 Assay kit. Experiments were performed in triplicate (*p < 0.05)

To determine if growth inhibition of feline OSCC cells was mediated by apoptosis, SCCF2 and SCCF3 were treated with DMSO, 0.02, 0.2, or 2 μM of LLL12 for 12 or 24 h and caspase 3/7 activity was measured. After 12 h of treatment with 0.2 μM of LLL12, SCCF2 showed a trend towards increased caspase 3/7 activity (data not shown), but this difference did not reach statistical significance until 24 h of treatment. SCCF3 showed a small, but statistically significant, increase in caspase 3/7 activity after 24 h of treatment with 2 μM of LLL12 (Fig. 4).

### Treatment with LLL12 reduces colony formation in feline OSCC cells

**Fig. 5:**
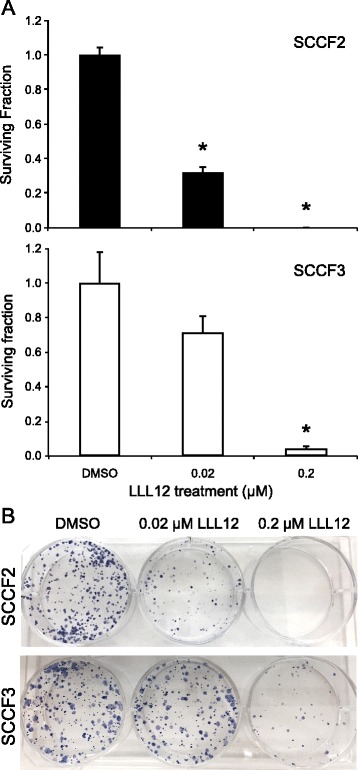
Impact of LLL12 on colony formation in feline OSCC cell lines. **a** Feline OSCC cells were seeded at 1,000 cells per well in 6 –well plates for 24 h, followed by treatment with DMSO, 0.02, 0.2 or 2 μM LLL12 until formation of visible colonies. Cells were then fixed and stained with crystal violet and colonies greater than 50 cells were counted. After counting colonies, plating efficiency and survival fraction were calculated. Plating efficiency was defined as the number of colonies formed divided by the number of cells seeded in DMSO treated groups. Survival fraction was defined as the number of colonies formed divided by the number of cells seeded in LLL12 treated groups, normalized to the plating efficiency (*p < 0.0001). **b** Photographs of representative 6-well plates

To further assess the effects of LLL12 on feline OSCC cell growth, SCCF2 and SCCF3 cells were seeded in 6-well plates and treated with DMSO or LLL12 and evaluated daily. Once colony formation was observed in DMSO treated wells, the plates were collected and colonies were counted following staining with crystal violet. As shown in Fig. 5, colony formation was significantly inhibited in both SCCF2 and SCCF3 at concentrations above 0.02 and 0.2 μM, respectively. Photographs of representative 6-well plates are shown in Figure 5b.

### Effects of LLL12 on STAT3 transcriptional targets

**Fig. 6:**
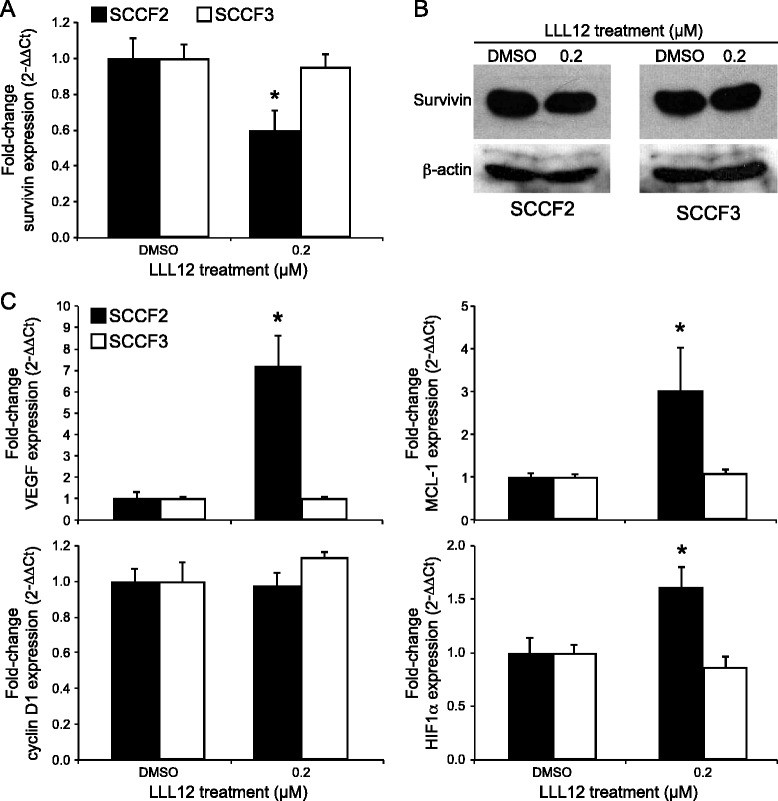
Effects of LLL12 on transcriptional targets of STAT3. Feline OSCC cells were treated with DMSO or 0.2 μM LLL12 for 12 h and RNA and protein were collected. Quantitative RT-PCR **(a)** and Western blotting **(b)** were performed to assess survivin mRNA and protein levels, respectively (*p < 0.0001). **(c)** Quantitative RT-PCR was performed to assess cyclin D1, MCL-1, VEGF and HIF1α mRNA expression in the cells treated above (*p < 0.05)

The effect of LLL12 on survivin, a transcriptional target of STAT3, was assessed by quantitative RT-PCR. Following 12 h of treatment, the expression of survivin was significantly downregulated in SCCF2 as demonstrated in Fig. 6a. A small decrease in survivin protein was also observed following LLL12 treatment as assessed by Western blotting (Fig. 6b). In contrast, no changes in survivin mRNA or protein levels were seen in SCCF3.

Quantitative RT-PCR was performed to evaluate the effects of LLL12 treatment on other STAT3 transcriptional targets, including cyclin D1, VEGF, and MCL-1. As shown in Figure 6c, there was no change in cyclin D1 gene expression in either cell line following treatment with LLL12. In contrast, there was an approximately 7-fold increase in VEGF gene expression in SCCF2 after LLL12 treatment. Likewise, expression of MCL-1, an anti-apoptotic protein whose transcription is regulated by both STAT3 and VEGF, was significantly increased in SCCF2 following LLL12 treatment. Finally, as HIF-1α is a known transcriptional regulator of VEGF activation, mRNA levels were also assessed by quantitative RT-PCR. As with VEGF and MCL-1, HIF-1α gene expression was significantly increased in the SCCF2 cell line following treatment. In contrast, no significant changes in VEGF, MCL-1 or HIF-1α gene expression were observed in the SCCF3 cell line.

### Activation of the PI3K/AKT/mTOR is not responsible for up-regulation of VEGF, MCL-1 and HIF-1α gene expression following LLL12 treatment

**Fig. 7:**
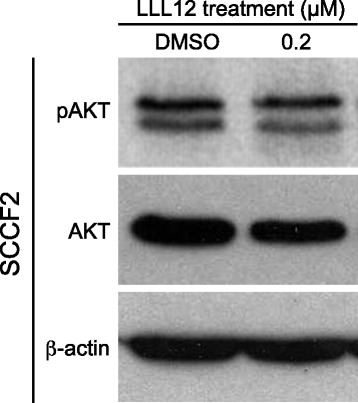
Evaluation of PI3K/AKT/mTOR signaling pathway in feline OSCC. Feline OSCC cells were treated with DMSO or 0.2 μM LLL12 for 12 h. Cells were harvested and total protein isolated followed by SDS-PAGE and Western blotting for pAKT, AKT and β-actin

To determine if activation of the PI3K/AKT/mTOR pathway is responsible for the observed up-regulation of VEGF, MCL-1 and HIF-1α gene expression in SCCF2 following LLL12 treatment, we evaluated the expression and phosphorylation status of AKT in these cells after 12 h of drug exposure. Western blotting demonstrated low levels of basal AKT phosphorylation in SCCF2, which remained unchanged after treatment with LLL12 (Fig. 7).

### Immunohistochemical expression of pSTAT3 in primary feline OSCC tumor samples

**Fig. 8:**
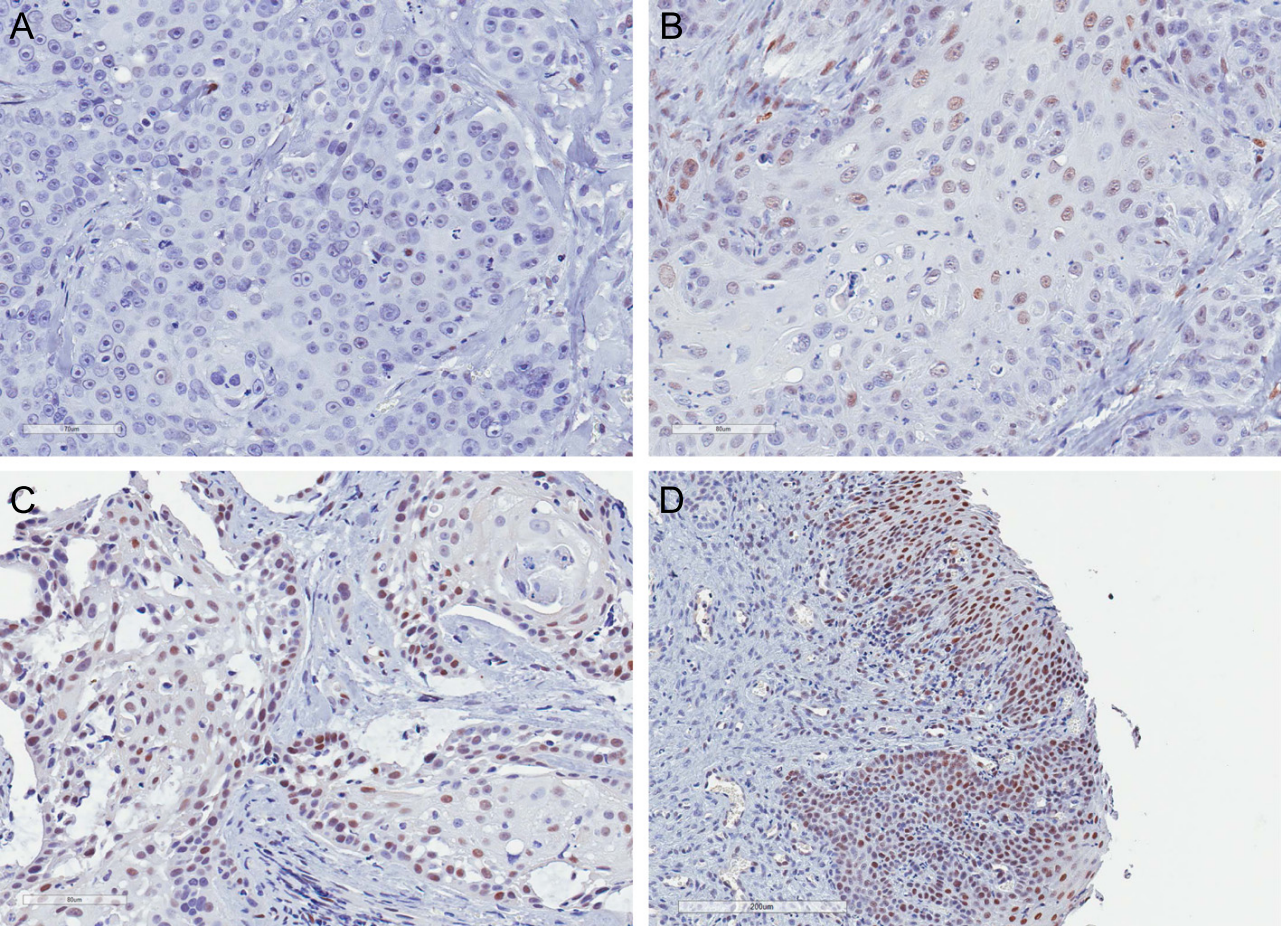
Immunohistochemistry for pSTAT3 in primary feline OSCC tumor samples. Immunohistochemistry was performed for pSTAT3 using a tissue microarray of feline OSCC tumor samples. Signal intensity and percent positivity were scored; these scores were added to generate a total score. Examples of no **(a)**, mild **(b)** and moderate **(c)** signal intensity are shown (20X). **d** pSTAT3 immunoreactivity was also observed in adjacent gingiva in 4 OSCC samples (10X)

To assess the prevalence of STAT3 activation in primary feline OSCC samples, a tissue microarray was constructed from 37 archived feline OSCC surgical biopsies and immunohistochemical staining for pSTAT3 was performed. Signal intensity and percent positivity were scored; these scores were added to generate a total score. Examples of no, mild and moderate signal intensity are shown in Figures 8a-c. Total scores of greater than or equal to 2 were considered positive. Of 37 samples, 27 had adequate tumor tissue for evaluation and 13 of these 27 (48.1 %) samples were considered positive. In 9 of the 13 positive samples, immunoreactivity was detected in 5–20 % of OSCC cells, while 3 and 1 sample displayed immunoreactivity in 20–50 % and 50–100 % of cells, respectively. In 4 samples, pSTAT3 expression was also observed in adjacent gingiva (Fig. 8d), although 2 of these 4 samples were considered to have inadequate tumor sample and were excluded from analysis. The results of the TMA scoring are summarized in Additional file 1: Table S2.

## Discussion

Feline oral squamous cell carcinoma is a devastating cancer, for which effective treatments are lacking. It shares several similarities with advanced or recurrent HNSCC, including tumor biology (both tumors are locally invasive and cause malignant osteolysis), molecular markers (EGFR, p53) and poor response of advanced stage disease to conventional therapies [36, 37]. In humans, key risk factors for development of HNSCC have been identified and include tobacco use, alcohol consumption and human papillomavirus (HPV) infection. In contrast, the etiopathogenesis of feline OSCC is not well understood. Although papillomavirus DNA has been isolated from feline cutaneous and nasal planum SCC [38, 39], no viral DNA was amplified in a series of 30 feline OSCC samples [40]. Retrospective, case-controlled studies have identified potential risk factors for development of feline OSCC, including use of flea control products, diet and exposure to environmental tobacco smoke [41, 42]. However, given the retrospective nature of these studies, a cause and effect relationship cannot be definitively determined.

Despite multiple clinical efforts to identify effective treatments for feline OSCC, few studies have investigated the molecular abnormalities that promote the development of this cancer and drive resistance to therapy. As with humans, activation of the epidermal growth factor receptor (EGFR) may be important as this has been shown to be widely expressed in feline OSCC tumor samples [43, 44] and EGFR inhibition by a small molecule inhibitor and RNA interference resulted in reduced proliferation and migration in a feline cell line [45]. However, dysregulation of other signaling pathways is poorly characterized.

In HNSCC, several potential contributors to disease pathogenesis have been identified including activation of STAT3 signaling. Specifically, phosphorylated STAT3 has been shown to be upregulated in both tumor and normal epithelium from HNSCC patients as compared to epithelium from non-cancer patients [12]. Furthermore, multiple studies have demonstrated that increased levels of activated STAT3 in patient tumor samples is associated with tumor stage, nodal metastasis and decreased survival [14, 46, 47], although conflicting reports also exist [48, 49]. Recently, a phase 0 clinical trial evaluating a STAT3 decoy oligonucleotide demonstrated decreased expression of STAT3 target genes in head and neck tumors following intratumoral injection [9]. Given the emerging role of STAT3 in human HNSCC, we elected to evaluate its potential function in the feline disease. Our collaborator (C. Lin, College of Pharmacy, OSU) has developed a small molecule, allosteric STAT3 inhibitor (LLL12) that exhibits broad activity against both human and canine cancer cell lines at concentrations in the nanomolar/low micromolar range [24, 50]. The purpose of this study was to characterize the expression of pSTAT3 in feline OSCC cell lines and tumor samples and to investigate the effects of LLL12 in feline OSCC cell lines.

Our initial work showed that one cell line, SCCF2, had high levels of pSTAT3 compared to the other two OSCC cell lines. Although treatment with LLL12 significantly reduced proliferation in all cell lines, SCCF2 was the most sensitive to drug exposure (IC_50_ = 180 nM), correlating with the highest level of basal pSTAT3. We further evaluated the effects of LLL12 on cell proliferation with colony formation assays in high and low pSTAT3 expressing cell lines, (SCCF2 and SCCF3, respectively). Colony formation was significantly reduced in both cell lines; however, this occurred at a drug concentration 10 times lower for SCCF2 (20 nM) compared to the SCCF3 line (200 nM), providing further support for STAT3 specific effects. In both cell lines, inhibition of colony formation occurred at concentrations well below the calculated IC_50_ values. While the exact reason for this discordance is not known, constitutive STAT3 phosphorylation has been previously associated with enhanced colony formation [51]. In contrast to our study, effects on colony formation with other STAT3 inhibitors generally occurred at drug concentrations approximating IC_50_ values [52, 53] suggesting that other factors (i.e. culture conditions, anchorage dependence, etc.) may have influenced the sensitivity of our feline OSCC lines to LLL12.

Treatment of SCCF2 with LLL12 resulted in downregulation of both pSTAT3 and STAT3 protein expression. Mechanisms of STAT3 protein downregulation include autoregulation of gene transcription and ubiquitin-mediated proteasomal degradation [33–35]. However, STAT3 mRNA levels were unchanged following LLL12 treatment and concurrent treatment with a proteasome inhibitor failed to reverse STAT3 downregulation, suggesting that neither of these mechanisms are responsible for the observed decreased in STAT3 protein. Other potential reasons for loss of total STAT3 include caspase-dependent proteolysis [54] and post-transcriptional modification by microRNAs [55].

Apoptosis of SCCF2 cells, as detected via caspase 3/7 activity, occurred at concentrations of LLL12 that reduced pSTAT3 expression, indicating that pSTAT3 inhibition may lead to apoptosis. One possible mechanism of apoptosis is downregulation of STAT3 transcriptional targets, which include anti-apoptotic genes. In support of this, survivin mRNA was significantly downregulated following treatment with LLL12; however, only a relatively small concomitant decrease in survivin protein was observed. This may be due to the prolonged presence of existing survivin protein, necessitating significant turnover before downregulation would be noted by western blotting, as previously described [50]. Importantly, SCCF3, a cell line with minimal basal pSTAT3, showed no change in survivin mRNA or protein levels, once again supporting a STAT3 specific effect.

To assess the prevalence of basal STAT3 phosphorylation in primary feline OSCC tissue samples, we constructed a TMA from archival formalin-fixed paraffin embedded tissue samples and performed IHC for pSTAT3. Based on our scoring criteria, 13 of 27 (48.1 %) samples that had adequate tissue for analysis were considered positive. This finding is consistent with previous HNSCC immunohistochemical studies that have demonstrated nuclear pSTAT3 immunoreactivity in 37–67 % of HNSCC tissue samples [14, 56]. It is difficult to compare studies due to differing methodologies employed, including those used to define STAT3 activation. In the current investigation we used nuclear accumulation of pSTAT3 as an indicator of activation. Other studies have relied on nuclear localization of unphosphorylated STAT3 or increased nuclear or cytoplasmic levels of unphosphorylated STAT3 as compared to control tissues [48, 57]. Interpretation of STAT3 activation is complicated by the discovery that unphosphorylated STAT3 can also enter the nucleus and activate gene expression via acetylation of other sites on the protein [58, 59]. Nevertheless, the presence of pSTAT3 in nearly half of the tumors examined indicates that this transcription factor is activated in a substantial proportion of feline OSCC.

Given the lack of complete patient information and follow-up accompanying the archival feline tumor samples, it was not possible to determine the prognostic significance of nuclear pSTAT3 immunoreactivity in feline OSCC. In 9 of the 13 samples that were considered positive, immunoreactivity was detected in less than 20 % of tumor cells. Reasons for this low percentage of positivity include tumor heterogeneity, sample aging and effects of and variation in fixation methods. Another possibility is that STAT3 activation is an early step in feline OSCC carcinogenesis and reliance on STAT3 signaling pathways is lost in later stages of tumor development. This theory is supported by HNSCC studies that have demonstrated increased STAT3 activation in normal mucosa from patients with HNSCC as well as in pre-malignant lesions such as leukoplakia [12, 14]. In addition, decreasing STAT3 activation with increasing tumor grade has also been observed [57]. As most cases of feline OSCC are diagnosed at an advanced stage, our results may support loss of STAT3 activation with tumor progression although further work is needed to investigate the expression of STAT3 in early tumorigenesis. Interestingly, pSTAT3 immunoreactivity was observed in adjacent mucosa in a handful of feline OSCC samples, which may further support the theory of early STAT3 activation.

One of the more intriguing findings in this study was the upregulation of expression of several STAT3 transcriptional targets, VEGF, MCL-1 and HIF-1α following LLL12 treatment. It is possible that the HIF-1α upregulation is responsible for the observed increase in VEGF and MCL-1, although the mechanism for HIF-1α up-regulation is not yet known. Moreover, several other studies have demonstrated downregulation of these targets following STAT3 inhibition [12, 25, 46, 50, 60], suggesting that feline OSCC may utilize different pathways to regulate the expression of these genes.

To further investigate the potential contribution of additional signaling pathways in feline OSCC, we assessed levels of pAKT/total AKT in the SCCF2 line. The PI3K/AKT/mTOR pathway regulates a diverse set of oncogenic processes including cellular survival, metastasis, angiogenesis and glucose metabolism. This pathway is of particular interest in HNSCC as it is frequently mutated in primary HNSCC tumors [61] and may represent a mechanism of resistance to anti-EGFR therapy [62]. Furthermore, studies on the involution of mammary gland epithelial cells have demonstrated a role for STAT3 in downregulation of PI3K-AKT signaling via STAT3 mediated expression of PI3K regulatory subunits [63]. We observed no upregulation of pAKT following LLL12 treatment, suggesting that STAT3 does not negatively regulate this pathway in feline OSCC and that enhanced AKT activation is not responsible for the observed increase in HIF-1α.

Signaling pathway cross-talk and redundancy may also help explain our unexpected results regarding VEGF, MCL-1 and HIF-1α expression. For example, studies have demonstrated that STAT3 regulates HIF-1α expression through multiple mechanisms including direct activation of gene transcription and promotion of protein stabilization [64–66]. In contrast, another study showed that HIF-1α, in cooperation with heat shock protein 90, regulates STAT3 activation and expression in colorectal cancer cells [67]. These findings underscore the complexity of STAT3 signaling pathways. Finally, cellular context may also influence the nature of STAT3 signaling. In contrast to its generally accepted role as an oncogene, a growing number of recent studies have demonstrated a growth suppressive function of pSTAT3 due to its activation of genes that inhibit tumorigenesis, including p21 and members of the FOX family of transcription factors [68]. This is further supported by work in tumor cell lines showing that STAT3 is a negative regulator of tumor growth in certain cancers such as thyroid carcinoma [69]. Although the results of our study are most supportive of growth stimulating role of STAT3 in feline OSCC, the upregulation of STAT3 target genes following LLL12 treatment indicate that STAT3 inhibition may be most effective when used in combination with other therapeutics.

## Conclusions

These data demonstrate that LLL12 has biologic activity against a feline OSCC cell line with constitutive STAT3 phosphorylation. In addition, pSTAT3 expression was detected in 48 % of feline OSCC tumor samples, further supporting STAT3 as a potential therapeutic target in this disease. However, LLL12 treatment also resulted in upregulation of several STAT3 transcriptional targets, suggesting that further research into the role of STAT3 in feline OSCC is needed.

### Availability of supporting data

All supporting data are included as additional files.

## Additional files

Additional file 1: Table S1. Primers for feline reverse transcriptase polymerase chain reactions. **Table S2.** Summary of STAT3 immunohistochemistry on feline OSCC tumor samples. A tissue microarray containing 37 feline OSCC tumor samples was constructed and immunohistochemistry for pSTAT3 was performed. Of the 37 tumor samples, 27 samples had enough tumor tissue for analysis. Overall pSTAT3 signal intensity of OSCC cells was subjectively scored from 0 to 3 (0 = none to weak, 1 = mild, 2 = moderate, 3 = strong), with strong reactivity defined as staining intensity of the positive control. The percentage of positive cells was also estimated and scored as follows: <5% = 0, 5–20% = 1, 20–50% = 2, 50–100% = 3. A total score was obtained by adding the score of signal intensity and percentage positivity. Total scores greater than or equal to 2 were considered positive.

## Abbreviations

EGFR: Epidermal growth factor receptor
HNSCC: Head and neck squamous cell carcinoma
JAK: Janus kinase
OSCC: Oral squamous cell carcinoma
PARP: Poly (ADP- ribose) polymerase
PIAS3: Protein inhibitors of activated STAT3
SHPs: SH2-containing phosphatases
STAT3: Signal transducer and activator of transcription 3
SOCS: Suppressors of cytokine signaling
