# Supplementary material for: Characterization of STAT3 expression, signaling and inhibition in feline oral squamous cell carcinoma
Source: BMC Vet Res. 2015 Aug 14;11:206. doi: 10.1186/s12917-015-0505-7 (PMC4536595; doi:10.1186/s12917-015-0505-7)
Supplement: Additional file 1: Table S1. — Primers for feline reverse transcriptase polymerase chain reactions. Table S2. Summary of STAT3 immunohistochemistry on feline OSCC tumor samples. A tissue microarray containing 37 feline OSCC tumor samples was constructed and immunohistochemistry for pSTAT3 was performed. Of the 37 tumor samples, 27 samples had enough tumor tissue for analysis. Overall pSTAT3 signal intensity of OSCC cells was subjectively scored from 0 to 3 (0 = none to weak, 1 = mild, 2 = moderate, 3 = strong), with strong reactivity defined as staining intensity of the positive control. The percentage of positive cells was also estimated and scored as follows: <5% = 0, 5–20% = 1, 20–50% = 2, 50–100% = 3. A total score was obtained by adding the score of signal intensity and percentage positivity. Total scores greater than or equal to 2 were considered positive. [file 12917_2015_505_MOESM1_ESM.docx]

**Table S1.** Primers for feline reverse transcriptase polymerase chain reactions

| **Primers** | **Primer Sequences** |
| --- | --- |
| **Feline cyclin D1 F** | 5’ – GTC TGC GAG GAG CAG AAG T – 3’ |
| **Feline cyclin D1 R** | 5’ – AGA GGA AGT GCT CGA TGA AGT – 3’ |
| **Feline HIF1α F** | 5’ – GAG TTC ACC TGA GCC TAC CAG – 3’ |
| **Feline HIF1α R** | 5’ – GCC GGC TGG AAT ACT GTA ATT GTG – 3’ |
| **Feline MCL-1 F** | 5’ – CAA CCA CGA GAC AGC CTT CCA AG – 3’ |
| **Feline MCL-1 R** | 5’ – CAC TGA AAA CAT GGA CAA TCA C – 3’ |
| **Feline STAT3 F** | 5’ – GGC CAT TGG TCA TCA AGA CTG– 3’ |
| **Feline STAT3 R** | 5’ – GCC GTT GTT GGA CTC TTC CAT G– 3’ |
| **Feline survivin F** | 5’ – GCT TCA TCC ACT GTC CCA CTG – 3’ |
| **Feline survivin R** | 5’ – GCA CAG CGC ACT CTC TTT GCA G – 3’ |
| **Feline VEGF F** | 5’ – GTC TAC CAG CGC AGC TAC TG – 3’ |
| **Feline VEGF R** | 5’ – GCC TTG CAA CGC GAG TCT GTG – 3’ |
| **Feline GAPDH F** | 5’ – GTC ATC CAT GAC CAC TTC GG – 3’ |
| **Feline GAPDH R** | 5’ – GAC AAC CTG GTC CTC AGT GTA G – 3’ |

**Table S2. Summary of STAT3 immunohistochemistry on feline OSCC tumor samples.** A tissue microarray containing 37 feline OSCC tumor samples was constructed and immunohistochemistry for pSTAT3 was performed. Of the 37 tumor samples, 27 samples had enough tumor tissue for analysis. Overall pSTAT3 signal intensity of OSCC cells was subjectively scored from 0 to 3 (0 = none to weak, 1 = mild, 2 = moderate, 3 = strong), with strong reactivity defined as staining intensity of the positive control. The percentage of positive cells was also estimated and scored as follows: <5% = 0, 5-20% = 1, 20-50% = 2, 50-100% = 3. A total score was obtained by adding the score of signal intensity and percentage positivity. Total scores greater than or equal to 2 were considered positive.

*Samples that demonstrated pSTAT3 staining in adjacent gingiva

| **Tumor Sample No.** | **Staining Intensity** | **% of cells** | **Total Score** | **Positive/ Negative** |
| --- | --- | --- | --- | --- |
| 1 | 1 | 1 | 2 | Positive |
| 2 | 2 | 1 | 3 | Positive |
| 3* | 1 | 1 | 2 | Positive |
| 4 | 1 | 0 | 1 | Negative |
| 5 | 1 | 0 | 1 | Negative |
| 6 | 1 | 0 | 1 | Negative |
| 7 | 0 | 0 | 0 | Negative |
| 8 | 2 | 3 | 5 | Positive |
| 9 | 1 | 0 | 1 | Negative |
| 10 | 0 | 0 | 0 | Negative |
| 11 | 1 | 1 | 2 | Positive |
| 12 | 2 | 1 | 3 | Positive |
| 13 | 0 | 0 | 0 | Negative |
| 14 | 1 | 0 | 1 | Negative |
| 15 | 2 | 2 | 4 | Positive |
| 16 | 1 | 2 | 3 | Positive |
| 17 | 1 | 0 | 1 | Negative |
| 18 | 2 | 1 | 3 | Positive |
| 19 | 1 | 1 | 2 | Positive |
| 20 | 2 | 1 | 3 | Positive |
| 21* | 2 | 1 | 3 | Positive |
| 22 | 2 | 2 | 4 | Positive |
| 23 | 0 | 0 | 0 | Negative |
| 24 | 1 | 0 | 1 | Negative |
| 25 | 1 | 0 | 1 | Negative |
| 26 | 1 | 0 | 1 | Negative |
| 27 | 1 | 0 | 1 | Negative |
